# Differentiation-dependent susceptibility of human muscle cells to Zika virus infection

**DOI:** 10.1371/journal.pntd.0008282

**Published:** 2020-08-20

**Authors:** Vincent Legros, Patricia Jeannin, Julien Burlaud-Gaillard, Thibault Chaze, Quentin Giai Gianetto, Gillian Butler-Browne, Vincent Mouly, Jim Zoladek, Philippe V. Afonso, Mariela-Natacha Gonzàlez, Mariette Matondo, Ingo Riederer, Philippe Roingeard, Antoine Gessain, Valérie Choumet, Pierre-Emmanuel Ceccaldi

**Affiliations:** 1 Unité Epidémiologie et Physiopathologie des Virus Oncogènes, Département de virologie, Institut Pasteur, Paris, France; 2 Université de Paris, Paris, France; 3 UMR CNRS 3569, Paris, France; 4 INSERM U1259 & Plate Forme IBiSA de Microscopie Electronique, Université François Rabelais and CHRU, Tours, France; 5 Proteomics Platform, Mass Spectrometry for Biology Unit, USR 2000 IP CNRS, Institut Pasteur, Paris, France; 6 Bioinformatics and Biostatistics Hub, C3BI, USR 3756 IP CNRS, Institut Pasteur, Paris, France; 7 Sorbonne Université, Institut National de la Santé et de la Recherche Médicale, Association Institut de Myologie, Centre de Recherche en Myologie, UMRS974, Paris, France; 8 Laboratory on Thymus Research, Oswaldo Cruz Institute, Oswaldo Cruz Foundation, Rio de Janeiro, Brazil; 9 Brazilian National Institute of Science and Technology on Neuroimmunomodulation (INCT-NIM), Rio de Janeiro, Brazil; 10 Unité Environnement et Risques Infectieux, Département de santé globale, Institut Pasteur, Paris, France; Fundacao Oswaldo Cruz, BRAZIL

## Abstract

Muscle cells are potential targets of many arboviruses, such as Ross River, Dengue, Sindbis, and chikungunya viruses, that may be involved in the physiopathological course of the infection. During the recent outbreak of Zika virus (ZIKV), myalgia was one of the most frequently reported symptoms. We investigated the susceptibility of human muscle cells to ZIKV infection. Using an *in vitro* model of human primary myoblasts that can be differentiated into myotubes, we found that myoblasts can be productively infected by ZIKV. In contrast, myotubes were shown to be resistant to ZIKV infection, suggesting a differentiation-dependent susceptibility. Infection was accompanied by a caspase-independent cytopathic effect, associated with paraptosis-like cytoplasmic vacuolization. Proteomic profiling was performed 24h and 48h post-infection in cells infected with two different isolates. Proteome changes indicate that ZIKV infection induces an upregulation of proteins involved in the activation of the Interferon type I pathway, and a downregulation of protein synthesis. This work constitutes the first observation of primary human muscle cells susceptibility to ZIKV infection, and differentiation-dependent restriction of infection from myoblasts to myotubes. Since myoblasts constitute the reservoir of stem cells involved in reparation/regeneration in muscle tissue, the infection of muscle cells and the viral-induced alterations observed here could have consequences in ZIKV infection pathogenesis.

## Author summary

Muscle cells are potential targets of many arboviruses, such as Ross River, Dengue, Sindbis, and chikungunya viruses, and may be involved in the disease manifestation. During the recent outbreak of Zika virus (ZIKV), myalgia was one of the most frequently reported symptoms. We investigated the susceptibility of human muscle cells to ZIKV infection. Using an *in vitro* model of human muscle stem cells (myoblasts) that can be differentiated into differentiated muscle cells (myotubes), we found that myoblasts can be infected by ZIKV. In contrast, myotubes were shown to be resistant to ZIKV infection. Infection induced the death of infected cells. Protein levels 24h and 48h post-infection indicate that ZIKV infection induces an upregulation of proteins involved in the activation of the Interferon type I pathway, and a downregulation of protein synthesis. This work constitutes the first observation of primary human muscle cells susceptibility to ZIKV infection, muscle stem cells being susceptible while differentiated muscle cells are resistant. Since myoblasts constitute the reservoir of stem cells involved in reparation/regeneration in muscle tissue, the infection of muscle cells and the viral-induced alterations observed here could have consequences during ZIKV infection.

## Introduction

Zika virus (ZIKV) is a mosquito-borne flavivirus transmitted by many *Aedes* species (*Ae*. *africanus*, *Ae aegypti*, *Ae*. *hensilli*, *Ae*. *luteocephalus*). ZIKV was first isolated in the Zika forest, near Entebbe in Uganda from a sentinel Rhesus monkey with an acute febrile illness in 1947 [1]. Up to 2007, cases of Zika infection were sporadically reported in Africa and South East Asia, and were associated with mild clinical symptoms. Thus, ZIKV has received very little attention when compared to other emerging arboviruses, such as Dengue, chikungunya, Japanese encephalitis or West Nile viruses. The first reported epidemics occurred on the Yap archipelago (Federated States of Micronesia) in 2007 [2] and in French Polynesia in 2013 with 28,000 infections in the first 4 months of the epidemic [3]. In May 2015, the first cases appeared in the northeast regions of Brazil, preceding the largest Zika virus outbreak in this country. Around 30,000 clinical cases have been reported in 2016, but the total number of individuals infected remains unknown and could reach one million cases. ZIKV has now spread throughout Central and South America and the Caribbean, as well as the United States [4].

The main clinical features of acute ZIKV infection are fever, rash, arthralgia, peri-articular edema, and myalgia [5]. The latter has been also reported associated with other arboviral infections (dengue, chikungunya and West Nile viruses) [6], [7], [8]. During the recent outbreaks, a clear association has been demonstrated between ZIKV infection and neurological syndromes. Congenital infection can lead to microcephaly in infected children [9], and ZIKV infection can be associated with some Guillain-Barré Syndromes [10], [11]. The viral tropism has been demonstrated *in vitro* in human skin cells [12], human cortical progenitor cells [13], microglia (resident macrophages), and other human and non-human cell lines, including neuronal, placental, colonic, hepatic and rhabdomyosarcoma cells [14]. Several candidate viral receptors, such as AXL, DC-SIGN, Tyro3, TIM-1, have been proposed [12], [15], [16], [17], although their role is still a matter of debate [18], [19]. ZIKV infection induces cytopathic effects in most cell lines [14], characterized by massive cytoplasmic vacuolization and paraptosis-like cell death [20].

Human muscle involvement during ZIKV infection is based on several observations. First, myalgia is a frequent symptom of infection, reported in 44% of 297 cases during the 2013–2014 outbreak in French Polynesia [21], 60% in Martinique’s 2015–2016 outbreak [22], as well as in four of eight infected patients returning from Suriname to French Guiana [23]. In addition, muscle involvement has been extensively described in other arbovirus infections: alphaviruses, such as Mayaro, chikungunya and Sindbis viruses, have been reported to cause myalgia and myositis in humans [24] [7], [25], [26], and flaviviruses, e.g. West Nile and dengue viruses, have been associated with myositis [6], [8] and rhabdomyolysis [27], [28]. Although the mechanisms of such myopathies may be indirect, related either to viral-induced dehydration/hypophosphatemia, or myocytotoxic cytokines, the probability of a direct muscle cell targeting by the virus may be hypothesized. Indeed, Sindbis, dengue and chikungunya viruses are able to infect human primary myoblasts *in vitro* [7], [29], [26]. Moreover, case-reports from human biopsies have demonstrated the infection of human myoblasts during chikungunya virus (CHIKV) infection [7] and the infection of human myotubes during dengue virus infection [30]. Regarding ZIKV, infection of a rhabdomyosarcoma cell line [14], and the presence of viral RNA in muscle samples from experimentally infected mice and monkeys [31], [32] have been reported. In the present paper, we have developed a human muscle cellular model of ZIKV infection, and demonstrated the susceptibility of human myoblasts, but not differentiated myotubes, to ZIKV infection, confirming it to be a valuable model for the investigation of the host factors involved during ZIKV infection. Myoblasts are proliferating mononucleated muscle precursors. *In vivo*, myoblasts are quiescent progenitor muscle cells. After a lesion, they are able to activate and differentiate in myofibers. Thus, they are the central element of myogenesis, muscle self-renewal and repair after a lesion or a stress. *In vitro*, once confluent, these precursors can differentiate in dedicated culture conditions (see Materials and Methods) into multinucleated myotubes. Both myoblasts and myotubes express desmin, but they can be identified by the number of nuclei per cell using Hoescht staining.

## Results

### Human myoblasts are susceptible to infection by different ZIKV strains

**Fig 1:**
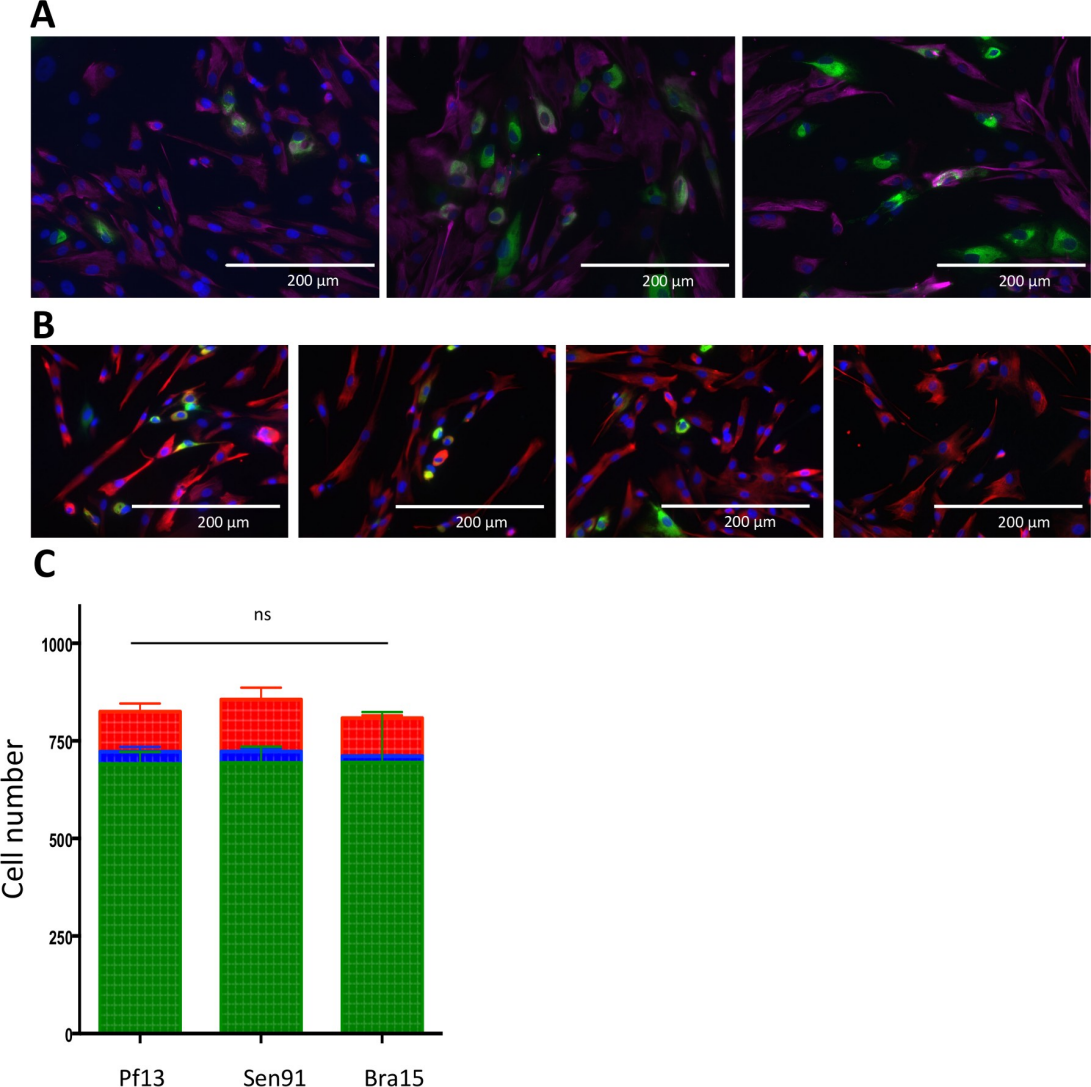
Infection of human myoblasts by ZIKV. A: Primary human myoblasts cells were infected with the Pf13 strain of ZIKV (MOI 1), and infection was assessed by immunofluorescence at different times p.i. (24, 48 and 72 h p.i., left, middle and right panels, respectively) using an anti-E antibody (4G2, green staining). Myoblasts were identified by desmin immunoreactivity, using a rabbit anti-desmin antibody (Dako; red staining) Nuclei are visualized by DAPI staining. Scale Bar: 200μm. B: Infection of human myoblasts at 48 h p.i., with three different ZIKV isolates (Pf13, Sen91 and Bra16, left, middle left and middle right panel, respectively) at a MOI 1. Right panel: Mock-infected control. Infection was assessed using an anti-E antibody (4G2) and myoblasts were identified by desmin immunoreactivity, using a rabbit anti-desmin antibody (Dako). Scale Bar: 200μm. C: Quantification of the percentage of ZIKV infected myoblasts. Human myoblasts were infected at a MOI 1, with three different ZIKV isolates (Pf13, Sen91, Bra16), and cell counting was performed on more than 750 cells from three different cultures, by assessing on immunofluorescence preparations (as described above), the number of cells immunoreactive for both E protein and desmin, i.e. the infected myoblasts (in red), those immunoreactive for E protein but not for desmin, i.e. infected non-muscle cells (in blue) and uninfected muscle/non muscle cells (in green). No significant difference was found between the three viral isolates (ns).

Although many human cell lines have previously been shown to be susceptible to ZIKV infection, little is known concerning ZIKV tropism to muscle cell. Therefore, we first established the susceptibility of primary human myoblasts to ZIKV (Pf13 strain, isolated from French Polynesia). At a multiplicity of infection (MOI) of 1, ZIKV envelope protein E immunoreactivity is detected at 24, 48 and 72h post-infection (p.i.) (Fig 1A). To identify potential strain specificity in terms of susceptibility, we performed the same analysis using two different ZIKV clinical isolates. Thus, similar immunoreactivity was also detected with two other ZIKV clinical isolates, Sen91 and Bra16, isolated from Senegal (1991) and Brazil (2016), respectively (Fig 1B). For all strains, 48 h p.i., around 15% of the primary myoblasts (i.e. desmine positive cells) were found to be immunoreactive (Fig 1C). Interestingly, a decrease in nuclear density was observed at late time-point but this was not correlated with typical observation.

### ZIKV induces a productive infection in human primary myoblasts from different donors

**Fig 2:**
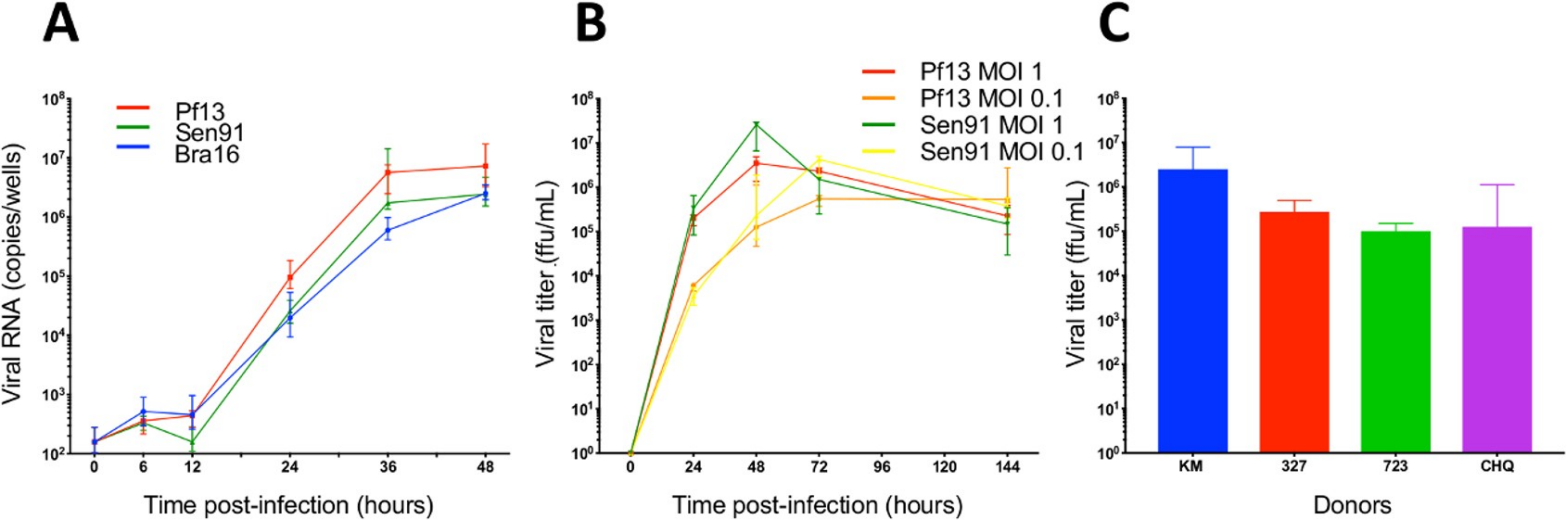
Different isolates of ZIKV induce a productive infection in primary human myoblasts, from different donors. A: Assessment of viral RNA production (number of copies/well, RT-qPCR) at 6, 12, 24, 36 and 48 h p.i. with infection by ZIKV isolates: Pf13 (red), Sen 91 (green), Bra 16 (blue), at a MOI 1. The dashed line corresponds to the detection limits. Results from three independent experiments performed in quadruplicates. B: Kinetics of viral titer on human primary myoblasts of two different ZIKV isolates (Pf13 and Sen91), at MOI 0.1 and 1 at 1, 2, 3 and 6 days p.i., assessed by viral focus-forming assay (expressed in ffu/ml). Red Curve: Pf13, MOI 1, Orange Curve: Pf13, MOI, 0.1, Green Curve: Sen91, MOI 1, Yellow curve: Sen 91, MOI 0.1. Results from three independent experiments performed in duplicates. C: ZIKV infection of human primary myoblasts from 4 different donors. Human primary myoblasts from four different donors (KM, 327, 723 and CHQ5B) were infected by ZIKV (isolate Pf13, MOI 1) and viral yielding was assessed by virus focus-forming assay. Results from one experiment performed in triplicates.

To characterize the replication properties of ZIKV in human myoblasts, cells were infected with the 3 ZIKV strains; infectious titers and viral RNA production were assessed at different times. As shown in Fig 2A, viral RNAs were detected at 24 h p.i. then increased up to 36 h p.i., with no significant differences between the 3 strains. We then quantified the production of infectious particles by myoblasts in the supernatant (ffu/ml). As early as day 1 p.i., infectious particles are detected in the supernatant of myoblasts infected with either strain, and the production of infectious particles reached a plateau between 2 or 3 days p.i. (Fig 2B). No significant differences in viral yielding could be found between the two strains (Fig 2B). To demonstrate that the response of the cells was not correlated to the donors, primary myoblasts isolated from 4 different donors were infected with the Pf13 strain at a MOI of 1. After 48 h p.i. infectious viral particles were detected in all cultures, ranging from 10^5^ to 3.10^6^ ffu/mL (Fig 2C).

### Susceptibility to infection is dependent on the differentiation state of the muscle cells

**Fig 3:**
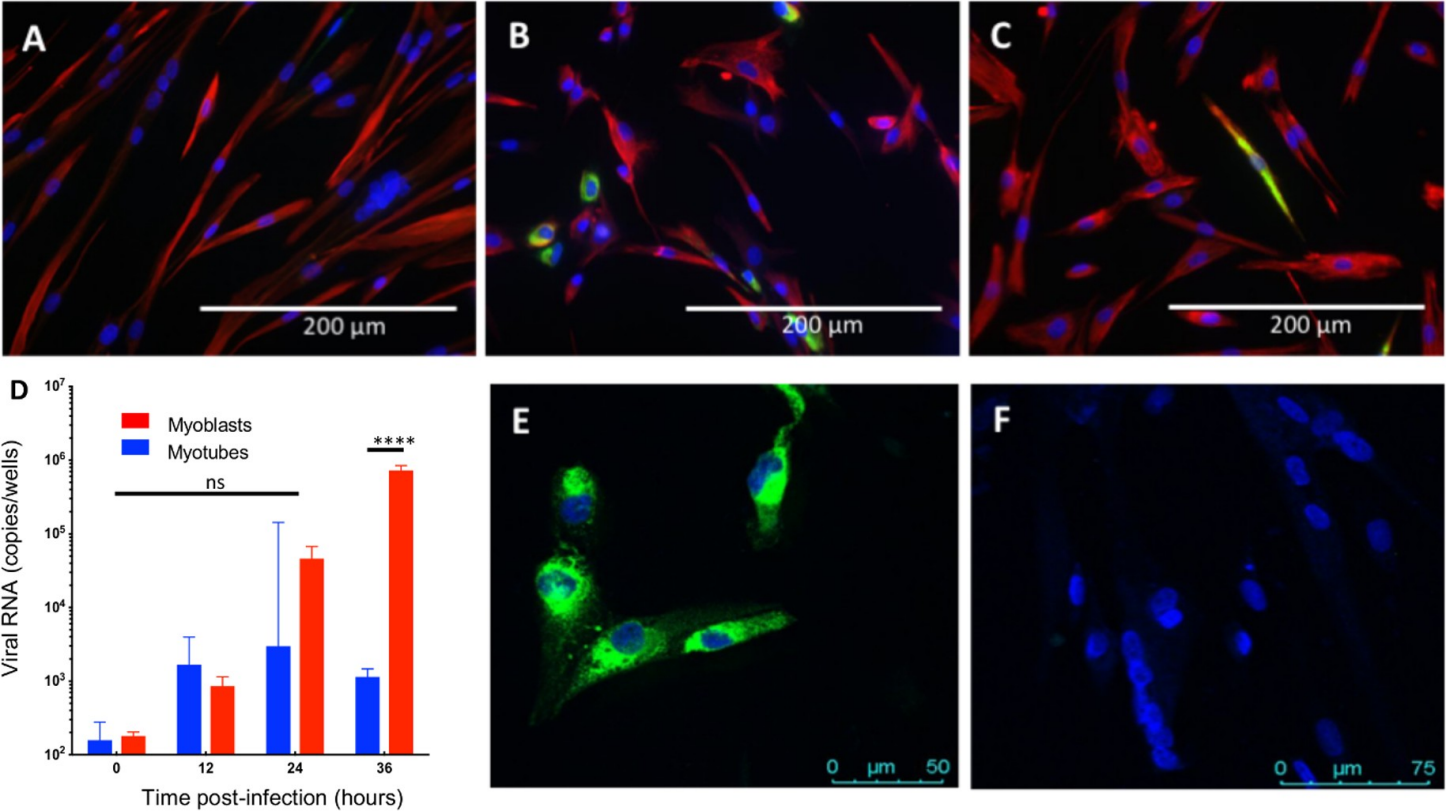
Infection of human myotubes and myoblasts by ZIKV. A: Human primary myoblasts (CHQ5B) were differentiated in DMEM medium for 7 days and infected with ZIKV (isolate Pf13, MOI 1). Replication curve (viral RNA) was assessed by RT-qPCR on the cell monolayer at 6, 12, 24 and 36 h p.i., in comparison to myoblasts. Results are expressed as median and interquartile range obtained from three independent experiments in quadruplicate (p-value<0.01, Mann Withney test). ****:p-value<0.0001 (Mann Whitney test). B: Primary human myotubes in DMEM medium, cells were infected with the Pf13 strain of ZIKV (MOI 1). Infection was assessed by immunofluorescence at 48 h p.i. using an anti-E antibody (4G2) (green); Myocytes were identified by desmin immunoreactivity, using a rabbit anti-desmin antibody (Dako) (red staining). C: The same experimental procedure with myoblasts infected in F10 medium (i.e.: medium for myoblasts). D: Same experimental procedure with myoblasts infected in DMEM medium (i.e.: medium for myotubes). Scale Bar: 200μm. E, F: Infection of the myogenic human cell line LHCN-M2, by ZIKV, MOI 1, 48 h p.i., as assessed by immunofluorescence using an anti-E antibody (4G2) (green) (E: Myoblast culture; F: Differentiated Myotube culture. Nuclei are visualized by DAPI staining. Scale Bar: 50 μm (E), 75 μm (F).

We then explored myotubes susceptibility to ZIKV infection. Myoblasts were cultured in F10 medium by DMEM (+ transferrin and insulin) and after six days, myotubes were considered as differentiated. While increasing amounts of viral RNAs were detected by RT-qPCR in infected myoblast cultures over time (6 to 36 h p.i.), low levels of viral RNAs were detected in myotubes, and limited increase could be observed after 12h p.i. (Fig 3A). Since some myoblasts always remain as reserve stem cells within the myotube cultures and could be responsible for such a viral RNA detection and great variance observed 24hpi, we confirmed by immunofluorescence the identity of infected cells. As shown in Fig 3C, viral immunoreactivity was detected only in the mononucleated myoblasts. It could never be detected in differentiated myotubes (multinucleated cells). Restriction of infection was not due to the change of the medium since myoblasts were found to be infected when maintained in differentiation medium for myotubes (DMEM) (Fig 3D).

Similar differential susceptibility was observed for other ZIKV isolates (Bra16, Sen91). Differences in susceptibility were also confirmed on the myogenic immortalized LHCN-M2 human cell line: myoblasts were found infected (as assessed by immunofluorescence) (Fig 3E) in contrast to differentiated myotubes (Fig 3F).

### Zika virus induces proteome changes in infected myoblasts

To decipher which cellular processes are affected during the infection of myoblasts by ZIKV, we measured the change in proteomes upon infection using a large-scale quantitative proteomic analysis by label-free quantification approach, with two different isolates, Pf13 and Sen91. We sampled infected and non-infected myoblasts at two different time-points. The first time-point was 24h p.i., a period that corresponds to the end of the first viral life cycles with an increasing viral replication. The second time-point was 48h p.i., a time at which the kinetics of replication reach a plateau. To ensure the robustness of the analyzes, we performed three independent biological replicates for each experimental condition. A similar number of proteins were identified from infected and uninfected cells. Around 4000 proteins were identified with good reproducibility between replicates in each condition (S1 Fig). Hierarchical clustering have been computed using the Jaccard index based distance. Samples clustered according to time post-infection, infectious/non infectious conditions and virus strains, respectively. Then, LFQ intensities of proteins from infected conditions were compared to the ones of uninfected conditions to highlight differentially abundant proteins. Results of the differential analyzes are summarized in S3 Fig.

### Pf13 infected cells vs mock

**Fig 4:**
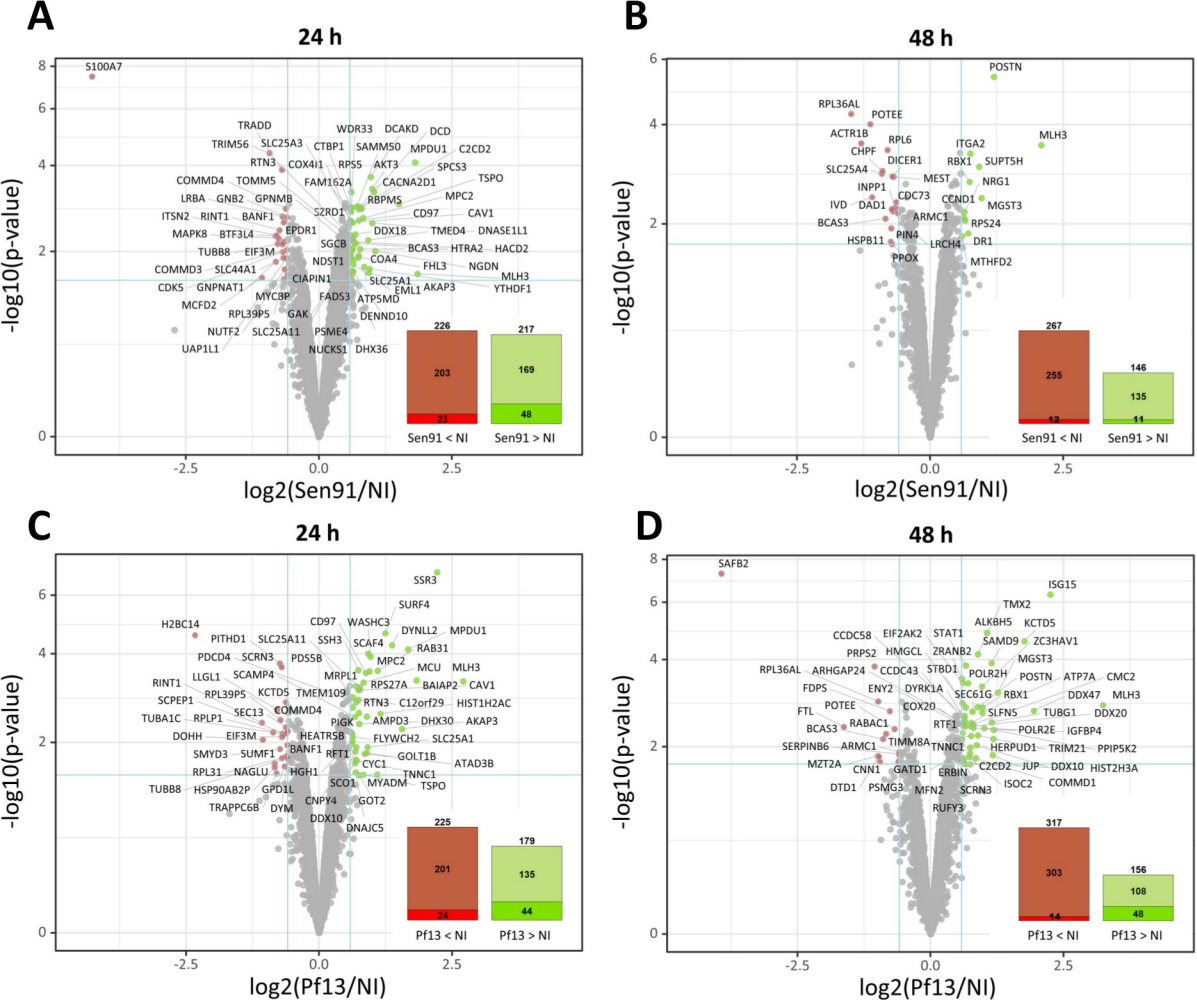
Proteomic analysis of ZIKV-infected myoblasts. Volcano plot graphs representing proteins differentially abundant between two biological conditions. Differential analyzes between human primary myoblasts infected with Pf13 or Sen91 Zika strains showing proteins differentially abundant. Each protein (represented as a dot) was mapped according to its log_2_ (fold change) on the abscissa axis and its–log_10_ (*t*-test *p*-value) on the ordinate axis. The proteins associated to an adjusted p-value inferior to an FDR level of 1% have been considered as significantly differentially abundant proteins. Mock versus Sen91 human primary myoblasts 24h and 48h post-infection (A, B). Mock versus Pf13 human primary myoblasts 24h and 48h post-infection (C, D). Red—proteins that are significantly less abundant in infected condition compared to mock-infected condition. Green—proteins that are significantly more abundant in infected condition compared to mock-infected condition. Grey—proteins that do not satisfy the fold-change and FDR cutoff.

**Fig 5:**
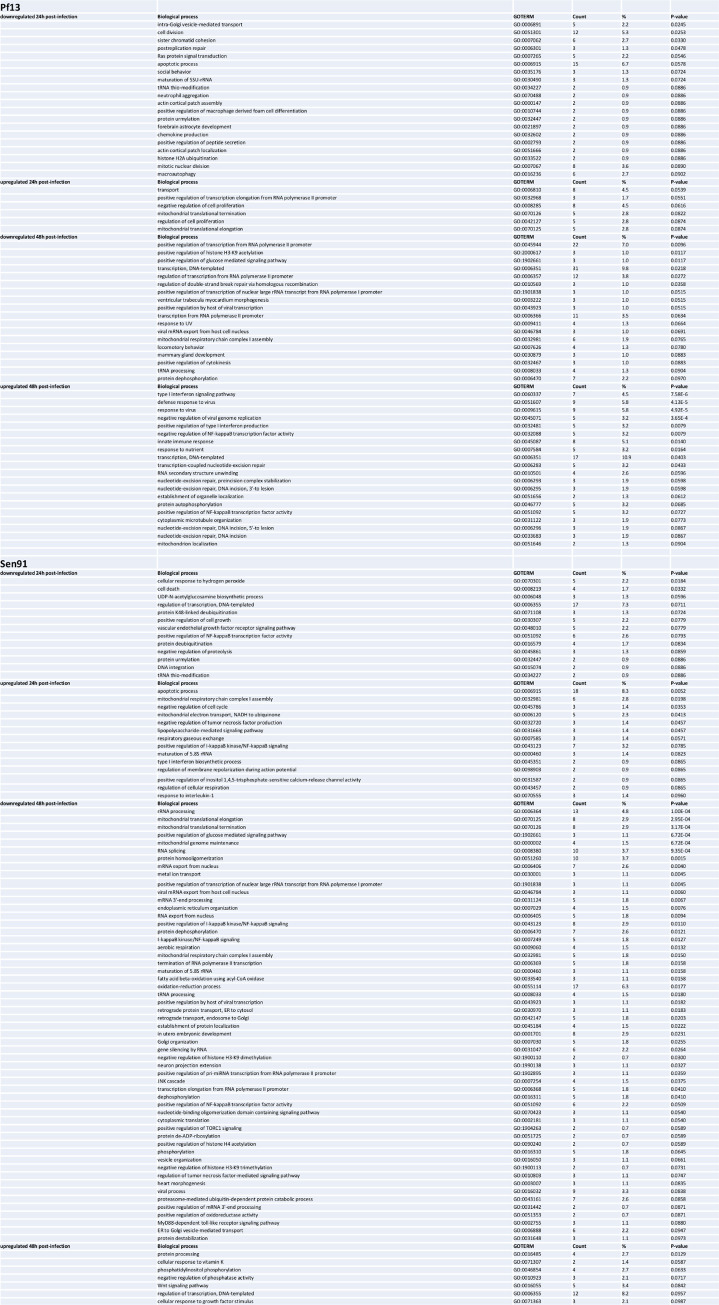
Enriched GO Biological Processes in ZIKV-infected myoblasts. Enriched GO Biological Processes among proteins modulated by Pf13 and Sen91 compared to mock-infected conditions 24h and 48h p.i (using DAVID software by selecting the set of quantified proteins among all replicates as background).

One day post-infection, 404 proteins were found differentially abundant between Pf13 and mock-infected cells, 179 of which are up-regulated in Pf13 while 225 are down-regulated (Fig 4C). Two days post-infection, a total of 473 proteins are modulated, 156 were up-regulated in Pf13 infected cells and 317 down-regulated (Fig 4D). Statistical enrichment tests were used to highlight major biological processes that may be affected during the infection. One day p.i., several biological processes are found enriched in infected cells compared to uninfected cells (Fig 5). “Transport” and “positive regulation of transcription” are enriched in up-regulated proteins while “intra-Golgi vesicle-mediated transport”, “cell division” and “post replication repair” are enriched in downregulated proteins. Two days post-infection, several GO terms associated with immune system processes are statistically enriched. More specifically, “type I interferon signaling pathway” and “negative regulation of viral genome replication” are found enriched in upregulated proteins, together with “negative regulation of NF-kappaB transcription factor activity”, “transcription-coupled nucleotide-excision repair” and “transcription, DNA-templated”. On the other hand, processes linked to protein expression are found enriched in down-regulated proteins, such as “positive regulation of transcription from RNA polymerase II promoter” and “positive regulation of histone H3-K9 acetylation”.

### Sen91 infected cells vs mock

One day post-infection, 217 are found upregulated in Sen91 infected cells when compared to mock and 232 downregulated (Fig 4A). Two days post-infection, 146 proteins are found more abundant and 273 less abundant in Sen91 infected cells (Fig 4B). When examining enriched biological processes, 8 biological processes are significantly enriched in Sen91 infected cells compared to uninfected ones at 24h p.i. Among them, “apoptotic process”, “mitochondrial respiratory chain complex I assembly”, “negative regulation of cell cycle”, “negative regulation of tumor necrosis factor production”, are enriched. “Cell death” is found enriched in downregulated proteins 24h p.i. Two days post-infection, one BP is found upregulated—“protein processing”—while 37 BPs are downregulated. “Mitochondrial genome maintenance”, “rRNA processing”, “I-kappaB kinase/NF-kappaB signaling”, “positive regulation by host of viral transcription” are found among them (Fig 5).

### Pf13 infected cells vs Sen91 infected cells

When comparing the proteins identified in Pf13 infected cells to the ones in the Sen91 infected cells, some BPs are similarly enriched regardless to the viral strain considered, mainly 48h p.i. Thereby, most of the BP found enriched in downregulated proteins following Pf13 or Sen91 infection are connected to host protein synthesis pathways. However, several BPs are found enriched in one condition compared to the other. One day p.i., “apoptotic process” is found enriched in protein more abundant in Sen91 infected cells than Pf13 ones, while “regulation of autophagy” is found in proteins more abundant in Pf13. Two days p.i., “type I interferon signaling pathway” is found upregulated only in proteins of Pf13 condition. All BPs changes are summarized in Fig 5.

### ZIKV infection of human myoblasts induces paraptosis cell death

#### Modulation of cell death processes

**Fig 6:**
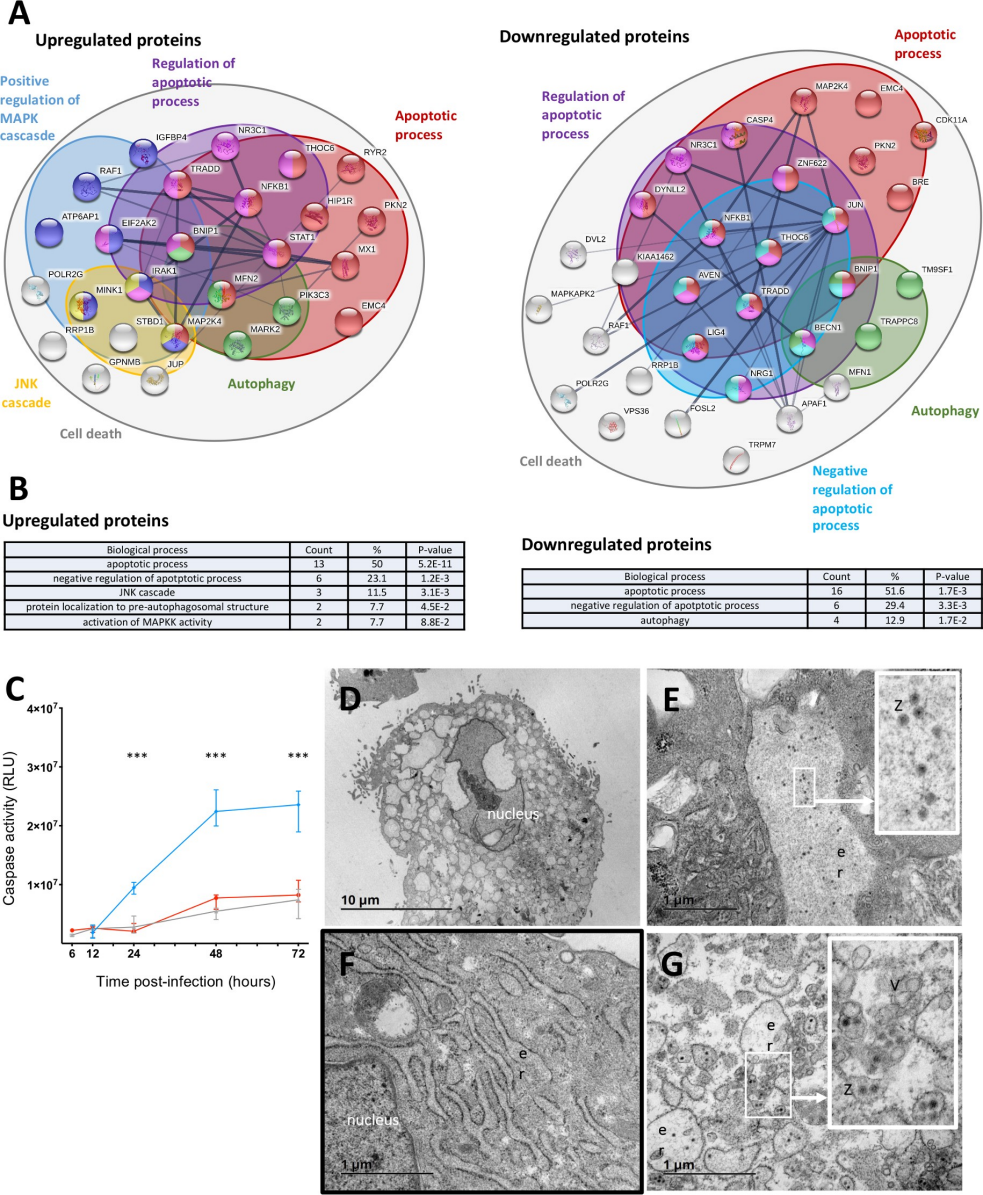
Exploration of ZIKV-induced cytopathic effects. Network representation (A) and gene ontology (B) of interacting proteins up-downregulated 48h after Pf13 infection compared to mock-infected (Up-downregulated proteins 48h after infection and involved in “Cell death”, “apoptotic cell death”, necrosis, autophagy, “necroptosis”, pyroptosis, MAP kinase cascade and JNK cascade were identified using Uniprot database. (A) Interaction analysis, STRING software; (B) Pathway enrichment analysis, DAVID software. (C) Caspase 3/7 activity measurement of mock-infected myoblasts (grey), ZIKV-infected myoblasts (red) and CHIKV-infected myoblasts (blue) at a MOI 1 (Promega Caspase-Glo 3/7 Assay kit). Results are expressed as median and interquartile range obtained from 4 independent experiments in triplicate. (D, E, F, G) Transmission Electron Microscopy was performed on mock (D) and infected (MOI = 1) myoblasts (B, C, E), 48h post-infection. er: endoplasmic reticulum, V: virus-induced vesicles, Z: ZIKV particle. Scale Bar: 10 μm (B), 1 μm (C,D,E).

Cellular processes linked to cell death have been specifically explored. Up-downregulated proteins 48h after infection and involved in “Cell death”, “apoptotic cell death”, necrosis, autophagy, “necroptosis”, pyroptosis, MAP kinase cascade and JNK cascade were identified using Uniprot database. Among upregulated proteins 48h after Pf13 infection, 26 are associated with at least one of the previous GO-term, 31 in down-regulated proteins. Interaction and enrichment analysis were performed using STRING [33] and DAVID [34] softwares (Fig 6). Our proteomic data indicate that many proteins involved in cell death mechanisms (either programmed or non-programmed) are deregulated after infection. More specifically, apoptosis seems to be the most regulated cell death mechanisms, since the “apoptotic process” was found to be enriched in up- (p-value = 5.2E-11) and down-regulated (p-value = 1.7E-13) proteins, together with “negative regulation of apoptotic process” (Fig 6B). Interestingly, none of the apoptotic effectors, such as caspase 3 and caspase 7 were found to be upregulated. On a proteomic level, no signature of another commonly described mode of cell death, such as “pyroptosis”, “necrosis” or “necroptosis” was found. Proteins related to “autophagy” were identified to be enriched within the data set of down-regulated proteins. Finally, the induction of paraptosis was explored through the regulation of MAPK/JNK and PI3K/Akt cascades, two pathways that have been described to induce paraptosis. Our proteomic data indicate that MAPK/JNK cascade is submitted to an early modulation, as shown by the up/down-regulation of many proteins involved in this pathway as early as 24h post-infection (S2 Fig). At 48 h p.i., “JNK cascade” and “activation of MAPKK activity” were also statistically enriched (p-value = 3.1E-3 and 8.8E-2), respectively) in the group of up-regulated proteins related to cellular death but not in the down-regulated proteins (Fig 6A and 6B). Interestingly, phosphatidylinositol 3-kinase catalytic subunit type 3 was upregulated at 48h p.i., while phosphatidylinositol 3-kinase regulatory subunit beta was downregulated, suggesting that the PI3K/A cascade is activated following ZIKV infection (S3 Fig). Two days after Sen91 infection, similar modulation was found at the proteome level, with “apoptotic process” being the more significantly enriched. This BP is found in upregulated proteins (4.2E-7) and downregulated proteins (p-value = 3.0E-11) together with “negative regulation of apoptotic process” (p-value = 6.8E-2 and p-value = 2.6E-3, respectively). Interestingly, caspase 7 is among upregulated proteins. An enrichment of “autophagy” is found in both upregulated (p-value = 6.7E-4) and downregulated proteins (p-value = 2.5E-4). As for Pf13, “necrosis”, “pyroptosis” and “necroptosis” were not found enriched. However, contrary to Pf13, “JNK cascade” and “positive regulation of MAPK kinase activity” (p-value = 7.5E-2) are found in enriched in downregulated proteins. “Activation of MAPK activity” is found enriched in upregulated proteins (p-value = 9.7E-2). Interestingly, as observed for Pf13 infection, Sen91 also upregulate phosphatidylinositol 3-kinase catalytic subunit type 3, and proteins involved in “phosphatidylinositol-mediated signaling” are found enriched after infection (p-value = 9.6E-2).

#### Induction of caspase independent cell death

Proteome changes observed after Pf13 infection indicated that apoptosis was the main modulated cell death pathway, together with autophagy and paraptosis. In order to evaluate the viral-induced cytopathic effect and to identify which programmed cell death was induced in myoblasts, we next assessed the activity of caspases 3/7 during infection. As shown in Fig 6C, infection of myoblasts by ZIKV did not induce significant changes between 6 and 72 h p.i. compared to non-infected controls, in contrast to CHIKV infection which was used as a positive control. The relatively low level of ZIKV infected myoblasts (approximately 15% 48h p.i.) could be responsible for the absence of detection of caspases activation. To ensure that apoptosis was not activated in infected cells, we performed a TUNEL labeling assay on ZIKV infected myoblasts. No staining could be observed, as for uninfected controls.

Analysis by transmission electron microscopy revealed no cytopathic cellular alterations commonly associated with apoptosis or autophagy, 48h post-infection. Instead, nuclear alterations, and a strong vacuolization process (Fig 6D), with endoplasmic reticulum membranes development and “vesicle packets” (Fig 6G) were observed. These observations are reminiscent of paraptosis, a caspase-independent form of cell-death. Viral particles could be detected in the lumen of the endoplasmic reticulum of infected cultures (Fig 6E). Interestingly, the endoplasmic reticulum lumen of infected cells appears to be less dense than in uninfected cells (Fig 6E and 6F, respectively), a feature which is characteristic of the shutting-off of cellular protein synthesis commonly observed during flaviviral infection. These results indicate that Zika virus modulation of programmed cell death revealed by the proteomic analysis does not lead to an apoptotic cell death but, rather, to paraptosis.

## Discussion

Following the recent outbreaks of ZIKV in the Pacific Ocean and Brazil, ZIKV infection has been shown to be associated in certain cases with neural alterations such as microcephaly in newborn infants and Guillain-Barré syndrome, and more commonly with acute symptoms that occur during the infection by other arboviruses such as fever, rash, arthralgia and myalgia. Muscle symptoms are a common feature of arbovirus infections since alphaviruses such as CHIKV and Ross River virus infection is accompanied by long-lasting arthralgia and myalgia [35], as well as flavivirus infections (dengue virus, West Nile virus) are accompanied by acute myalgia [36], [37]. In addition, muscle cells have been identified as target cells for infection by many arboviruses, such as CHIKV [7], [38], [39], Sindbis virus [26], Ross River virus [40] or dengue virus [30], [29]. Concerning ZIKV, several lines of evidence pointed out the involvement of muscle tissue prevalence of myalgias during infection, ranging from 44 to 60% in human cases [2], [22]), and experimental infection studies on mouse and monkey models revealed the presence of viral copies or infectious virus in muscular tissue at different times p.i., even for one study at day 35 p.i. [31], [41], [32]. In the present paper, we demonstrate the susceptibility of primary human myoblasts to ZIKV infection. This susceptibility seems to be a general feature of ZIKV infection since it was observed with three different field isolates from Africa, French Polynesia and Brazil, and with primary myoblasts from three different donors, as well as with the human muscle cell line LHCN-M2. Susceptibility of primary human myoblasts has been already described for CHIKV [7], [39], and Sindbis virus [26]. Although a low number of myoblasts were found infected, consistent viral yielding could be detected, raising the possibility that muscle cells could constitute a site of viral replication during ZIKV infection.

Interestingly, differentiated primary human myotubes were shown to be resistant to ZIKV infection. These data are in accordance with previous observations made during CHIKV infection, either in human primary muscle cells [7], in murine model [38], as well as in human muscle biopsies from CHIKV infected individuals [7]. Such an observation has also been reported for another alphavirus, i.e. Sleeping Disease Virus (SDV), that infects salmonids. In this model, primary cultures of trout myoblasts could be infected by SDV, but myotubes were shown to be resistant to infection as demonstrated *in vivo* [42]. This characteristic does not seem to be a general feature of virus infection of muscle cells since previous reports from our group with different collaborators indicate that human myotubes can be infected by Sindbis virus [26] and influenza viruses [43], at least *in vitro*. This raises the possibility that differentiation modifies the host determinants for muscle cell susceptibility to ZIKV infection, and opens new perspectives in the differentiation-dependent susceptibility towards infection. One of the simplest explanation for such a difference in sensitivity would be the presence of a receptor governing viral infection at the cell surface of myoblasts and its absence at the cell surface of myotubes. Using western-bot analysis, we compared the profiles of AXL, a candidate receptor for ZIKV, but no significant difference in expression could be shown between myoblasts and myotubes, suggesting that difference in sensitivity is not linked to AXL expression. More in-depth investigations are required to understand the differentiation-dependent susceptibility to ZIKV infection. In this way, proteomic studies illustrating the different changes that occur during human muscle cell differentiation, as already performed [44] could constitute the first step to assess the potential virus receptors or host cell restriction factors that govern muscle cells susceptibility to ZIKV infection during differentiation. According to Le Bihan et al., at least 243 proteins are modulated during myoblasts differentiation into myotubes [44].

To decipher the molecular pathways that are potentially associated with ZIKV infection, a proteomic study was performed on Pf13, Sen91 and mock-infected myoblasts. Protein modulation described here should be analyzed as a reflect of complex tissue level modification more than individual cells. In our study, proteome changes reflect protein modulation occurring in a population of infected and uninfected cells. Assigning proteome modulation to infected cells alone would be only speculation, in particular when considering the 15% infection rate, 48h post-infection. On the proteome level, interferon type I signaling pathway and apoptosis are the two main BPs that are found enriched in proteins upregulated in infected condition. This observation is consistent with the well-documented cell response following in the late stage of flaviviral infection, i.e. induction of type I interferon and cell death [45]. However, the infection seems to modulate protein synthesis process and energy metabolism as “rRNA processing” and aerobic respiration” are found downregulated and “mitochondrial respiratory chain complex assembly” and “protein processing” are found upregulated, which may reflect complex regulation of these BPs. This analysis, aggregating data from both viral strains, can be useful for identifying Zika virus signature on the proteome level but can also fail to unveil strain specific modulations. To get further insights into specific strain modulations, proteins from Pf13 and Sen91 conditions were compared and enriched BPs were identified. Pf13 or Sen91 differentially modulates many different proteins which could indicate a strain-specific effect on the proteome. One day p.i., Sen91 seems to specifically induce apoptosis while Pf13 infection regulates autophagy. This observation can be related to the slightly faster replication rate of Sen91 in myoblasts which could lead to an earlier activation of apoptosis in Sen91 infected myoblasts, since apoptosis is also found activated in Pf13 infected myoblasts two days p.i. More importantly, type I interferon signaling pathway proteins display a higher activation 48h p.i. in Pf13 infected myoblasts compared to Sen91 infected myoblasts. In human primary astrocytes, it has been shown that Sen91 strain infection results in a delayed activation of innate immune response genes compared to Pf13 strain [46]. This differential activation of type I interferon between Sen91 and Pf13 strains can be a reflection of cell response toward different kinetics of infection. This hypothesis is supported by the upregulation of type I interferon in Sen91 myoblasts two days after infection. Two days post-infection, the activation of type I interferon production would just start in Sen91 infected myoblast, while Pf13 infected myoblasts would already display an activation of type I interferon pathway.

We next focused our attention on the Pf13 strain. One day after infection, enrichment analysis reveals that Pf13 infection upregulates transport and transcription (S2 Fig), which are commonly observed features in the first stages of flaviviral infection [47]. Two days after infection, our proteomic data indicate that Pf13 infection of myoblasts results in the induction of type I interferon production, inhibition of viral replication and DNA repair mechanisms. These upregulated biological processes are similar to those described by Garcez et al. in ZIKV-infected neurospheres probably reflecting shared modulation mechanisms between these two cells types [48]. More precisely, IFIT-1, 2 and 3, IRAK1, ISG15 and STAT1 are found upregulated in our data set. IRAK1 excepted, these proteins have been previously identified as upregulated during ZIKV infection [46], [49]. On the other hand, we identified potential cellular pathways which were inhibited by Pf13 infection such as NF-kappaB transcription factor activity and the glucose signaling pathway. Finally, we showed that proteins involved in transcription processes are up-regulated while others are down-regulated, which can be the consequence of complex and opposite regulations induced by viral infection and host innate response. Infection of myoblasts is associated *in vitro* with a decrease in nuclear density, suggesting ongoing cell death. Interestingly, among the described mechanisms commonly involved in *Flavivirus* infection related to cell death, apoptosis and paraptosis appear to be the most modulated at the proteomic level. Paraptosis is a slower cell death mechanism than apoptosis whose induction does not rely on caspase activity, unlike apoptosis [50]. Morphologically, it is characterized by extensive vacuolization that results from the dilatation of the ER. The mechanisms associated with the activation of paraptosis activation is yet only poorly understood. However, studies showed that paraptosis can be mediated by the phosphoinositide 3‐kinase (PI3K), the mitogen-activated protein kinase (MAPK) kinase, MEK-2, and Jun *N*-terminal kinase (JNK) [51], and by expression of insulin‐like growth factor I receptor (IGF‐IR) [50], [52]. At the level of the proteome, many proteins involved in the MAPK cascade are found to be up and down-regulated by Pf13 infection (S2 Fig). The PI3K catalytic subunit (uniprot: Q8NEB9) is upregulated at 48 hours. Moreover, PIK3R2 (uniprot: O00459) the PI3K regulatory subunit ß, is found downregulated. High levels of PIK3R2 have been shown to be correlated with an inhibition of PI3K cascade [53]. Together, these results suggest an activation of MAPK and PI3K cascades, which are, among other effects, associated with the initiation of paraptosis cell death. Interestingly, none of the main apoptosis effectors such as caspase 3 and 7 seems to be upregulated on the proteomic level. The absence of such activation was confirmed by measurement of caspase 3/7 activity after ZIKV infection, in comparison with CHIKV infection, an alphavirus known to induce apoptosis in myoblasts [54]. Moreover, to check that the absence of caspase 3/7 activation is not due to the sensitivity of the measure related to a low infectivity rate, TUNEL assays were performed. No TUNEL signal has been observed in infected cells, 24 and 48 hours’ post-infection, demonstrating the absence of apoptosis induction at a MOI 1 in our conditions. To have further insight into ZIKV cell death mechanisms, cytopathic effects were observed using Transmission Electron Microscopy. No cytopathic cellular alterations commonly associated with necrosis, apoptosis or autophagy could be observed. Instead, “vesicle packets” and ER dilatation, common features associated with flaviviral infection, were frequently observed. This observation is in line with the upregulation of homocysteine-responsive endoplasmic reticulum-resident ubiquitin-like domain member 1 protein (Herpud 1, uniprot: Q15011) in infected myoblasts (S3 Fig). This protein is involved in ER stress response and its expression is induced by unfolded protein response (UPR). Kny et al. suggested that high levels of Herpud1 protected the cell from ER stress response-mediated cell death [55], and it has been recently described as an inhibitor of apoptosis [56]. Strikingly, infected cells display intense cytoplasm vacuolization, a hallmark of paraptosis-induced alterations. Paraptosis cell death has been linked to ZIKV infection in both primary cultures and cell line [20] and downregulation of PI3K/Akt pathway by AR-12 was recently shown to Zika virus replication [57]. Whether paraptosis is a general feature of ZIKV-infection or is restricted to specific cell types in which apoptosis would be inhibited remains unclear. In muscle tissue, apoptosis is generally inhibited and cell death occurs through a necrosis, which plays an important role in muscle regeneration [58]. We can hypothesize that inhibition of apoptosis in myoblasts allows paraptosis to occur, which correlate with the slow cell death rate observed in infected myoblasts cultures.

Altogether, the present data indicate that primary human myoblasts constitute a target for ZIKV infection. Such data are in agreement with the muscular symptoms that are observed during ZIKV infection [2], [59], [60], and with the muscular tropism of ZIKV during acute infections in murine models [31], [41], as well as in long-term persistence of ZIKV in a simian model. The differential susceptibility towards infection of myoblasts vs myotubes raises the question of the differentiation-associated factors that may be associated with such a susceptibility, and opens new ways in searching for potential virus receptors and/or restriction factors that occur during differentiation. Myoblasts constitute the progenitor cells in the muscular tissue; infection of stem cells has already been reported for ZIKV, since this virus has been reported to be able to infect neural progenitor cells [61], [62], [63], [64] with possible consequences in the neurological development disorders such as microcephaly. In this way, this work provides new lights as to the possible involvement of myoblast infection by ZIKV in the acute phase of the disease, i.e. as a primary site of replication, involvement in myalgia process, as well as for long-term consequences, i.e. persistence in tissues, as shown in the monkey [32] or alterations in muscle regeneration processes. As myoblasts constitute the reservoir of progenitor cells involved in the regeneration of muscle tissue, the results presented here indicate that myoblasts could constitute a valuable model for host factors involved during ZIKV infection.

## Methods

### Ethics Statement

Cells were provided by the AFM Tissue Bank (Paris, France). Written informed consent was obtained from all adult subjects or from parent’s child prior to the tissue being donated to MyoBank, a member of the EuroBioBank network, in accordance with the French legislation on bioethics, and with the institutional approval of the Pitié-Salpêtrière Hospital (Paris, France) with authorization numbers AC-2013-1869 and AC-2019-3502.

### Cells and culture conditions

Primary human muscle cells (i.e. adult muscle cells that persist in postnatal and adult muscle) were originally isolated from the quadriceps of a 5-day-old infant as previously described (‘‘CHQ5B”cells)[65]. Three other primary muscle cell cultures (called ‘‘AB327C17”, ‘‘AB723C15” and ‘‘KM45C19”) from three other donors, were also used in one experiment to test the susceptibility to ZIKV infection. CHQ5B cells, cultivated in Ham’s F-10 supplemented with 50mg/ml of gentamycin and 20% fetal bovine serum (FBS), were trypsinized when they reached half confluency. Differentiation of satellite cells into myotubes was induced for 7 days in DMEM medium supplemented with 100mg/ml transferrin, 10 mg/ml insulin and gentamycin. ‘‘AB327C17”, ‘‘AB723C15” and ‘‘KM45C19” were cultivated in DMEM supplemented with 25% 199 medium + 20% FCS + fetuin (25μg/mL) + ßFGF (0.5ng/mL) + EGF (5ng/mL). For some experiments, a human myogenic cell line, “LHCN-M2”, immortalized by expression of telomerase and cyclin-dependent kinase 4 was used as previously described [66].

### Viruses

Three different low passage ZIKV strains were used in this study. Asian lineages Pf13 (GenBank: KX369547.1) and Bra16 (GenBank: KU991811.1) or in cell lines experiment the ZIKV isolate Rio-U1, African lineage Sen91 (GenBank: KF383039.1). All strains were amplified through a limited number of passages on C6/36 cells.

### Cells infection

For infections, cells were seeded in 24-well tissue culture plates in culture medium. Aliquots of virus were diluted at the appropriate MOI (0.1, 1, 10) in 200μl of medium supplemented with 5% FCS and added to the cells. Plates were incubated for 2 hours at 37°C. Unadsorbed virus was removed and then 1 ml of culture medium supplemented with 10% FCS was added to the cells, followed by incubation at 37°C until collection.

### Virus titration

Viral focus-forming assays were performed on VERO E6 cells. Briefly, Vero E6 cell monolayers (1 × 10^5^ cells/well) in 24-well tissue-culture trays were inoculated in duplicate with 200μL 10-fold serial dilutions of each sample and incubated for 2 hours at 37 °C. Unadsorbed virus was removed, after which 1 ml of DMEM supplemented with 1.6% carboxymethyl cellulose (CMC) 2% FCS was added to each well and incubated for 3 days. The CMC overlay was aspirated, and the cells were washed with PBS and fixed with 4% paraformaldehyde for 15 min, followed by permeabilization with 0.1% Triton X-100 for 3 min. After fixation, the cells were washed with PBS and incubated for 1 h at room temperature with anti-E antibody (4G2), followed by incubation with HRP-conjugated anti-mouse IgG antibody. The assays were developed with the Vector VIP peroxidase substrate kit (Vector Laboratories) according to the manufacturer's instructions. The viral titers were expressed in focus-forming units (ffu) per milliliter.

### Viral replication

Total RNA from cells was extracted using the Nucleospin RNA II kit (Macherey-Nagel) according to the manufacturer's instructions. RNA was eluted in 70 μl of RNAse-free H_2_0. Each sample was analyzed in duplicate against a standard curve produced from a specific concentration range of synthetic RNA (NS5 sequence).

One-step reverse transcription quantitative PCR (RT-qPCR) was performed using the Power Sybr Green RNA-to-Ct one-step kit (Applied Biosystems). ZIKV primers were selected in the NS5 protein coding region: sense ZIKV/NS5/9318 (aagtacacataccaaaacaaagtg), anti-sense ZIKV/NS5/9419 (tccgctccccctttggtcttg).

RT-qPCR was performed using Applied Biosystem’s Fast Real-Time PCR 7500 System with the v.2.0.1 7500 software. The thermal cycling conditions comprised: a reverse transcription step at 48°C for 30 min, an inactivation step of RT/RNAse enzyme at 95°C for 10 min followed by 40 cycles of 95°C 15s and 60°C 1min, a final denaturation step where the temperature was increased from 60°C to 95°C during 20min and a step of 15s at 95°C.

### Immunofluorescence

Cells were grown on coverslips, infected with different MOI (0.1, 1, 10) and regularly fixed with 4% paraformaldehyde for 15 min at 37°C, then permeabilized with 0.1% Triton X-100 for 3 min. After fixation (paraformaldehyde 4% in PBS), the cells were washed with PBS and incubated for 1 h at room temperature with anti-E antibody (4G2, R&D Biotech), followed by incubation with a fluorophore-conjugated secondary antibody (Jackson Laboratories). For some experiments, anti-desmin antibody (AbCam) was used together with a different fluorophore-conjugated secondary antibody (Jackson Laboratories). The coverslips were mounted with ProLong gold antifade reagent with DAPI (Life Technologies). The slides were examined using a fluorescence microscope (EVOS).

### TUNEL apoptotic and caspase 3/7 activity assay

CHQ5B cells were infected with ZIKV strain Pf13 at MOI of 1. After infection, ZIKV-infected cells were processed at different time-point in accordance with manufacturer’s instructions (Promega Caspase-Glo 3/7 Assay kit) to determine caspase-3 activity. Luminescence of each sample well was analyzed in a plate-reading luminometer. The activity of caspase 3 was expressed as relative luminescence unit (RLU).

A terminal deoxynucleotidyl transferase (TdT) dUTP nick-end labeling (TUNEL) assay was performed using the *In Situ* Cell Death Detection Kit, Fluorescein (Roche), according to the manufacturer’s instructions. Briefly, 5.10^4^ CHQ5B cells were seeded in 24-well tissue-culture trays. Cells were infected with ZIKV strain Pf13 at an MOI of 1 for 2 hours at 37 °C. Infected cells were subsequently fixed 24h and 48h p.i. with 4% paraformaldehyde/PBS and endogenous peroxydase activity was blocked using 3% H_2_O_2_ in methanol for 10 min, at RT. Then, cells were permeabilized with 0.1% Triton-X/0.1% sodium citrate solution. Negative (without TdT) labeling controls were included, along with mock-infected controls. Samples were then subjected to labeling with the TUNEL reaction mixture prior to imaging analysis. Coverslips were mounted with ProLong gold antifade reagent with DAPI.

### Transmission Electron Microscopy

Human primary muscle cells were fixed for 24h in 4%PFA and 1% glutaraldehyde (Sigma) in 0.1 M phosphate buffer (pH 7,2). Cells were then washed (PBS) and post-fixed with 2% osmium teteroxide for 1h. Cells were then dehydrated in ethanol solutions of growing concentrations and propylene oxide. Samples were impregnated with a mixture propylene oxide/Epon resin (Sigma), (1:1), and left overnight in pure resin. After embedding, polymerization was allowed for 48 h at 60°C. Ultra-thin sections (70 nm) with a Leica EM UC7 ultramicrotome. Sections were stained with 5% uranyl acetate (Agar Scientific) and 5% lead citrate (Sigma), and observed with a JEOL 1011 transmission electron microscope.

### Protein extraction

CHQ5B cells were infected with ZIKV strain Pf13 or Sen91 at a MOI of 1. 24 hours and 48 hours p.i., cells were trypsinized, transferred into 1.5mL Eppendorf tube and centrifuged at 500G for 5 min at 4°C. Cells were resuspended in 100μL of 8M urea (pH 7.5) and stored at -80°C. Cells were subjected to ultrasound (Cup Horn, Sonics & Material) for 18 min at 4°C, with 2 sec pulse on and 2 sec pulse off, at the maximum amplitude. Samples were then centrifuged at 14.000G for 10 min at 4°C. Supernatants were collected and stored at -80°C before proteomic analysis.

### Proteomic analysis

#### Digestion of proteins

Protein samples were solubilized in urea 8 M, Tris 100 mM pH7.5, then Disulfide bonds were reduced with 5 mM tris (2-carboxyethyl)phosphine (TCEP) for 20 min at 23°C and alkylated with 20 mM iodoacetamide for 30 min at room temperature in the dark. Subsequently, LysC (Promega) was added for the first digestion step (protein to Lys-C ratio = 80:1) for 3h at 30°C. Then the sample was diluted to 1 M urea with 100 mM Tris pH 7.5, and trypsin (Promega) was added to the sample at a ratio of 50:1 for 16h at 37°C. Proteolysis was stopped by adding 1% formic acid. Resulting peptides were desalted using Sep-Pak SPE cartridge (Waters) according to manufactures instructions.

#### LC-MS/MS of tryptic digest

LC-MS/SM analysis of digested peptides was performed on an Orbitrap Q Exactive Plus mass spectrometer (Thermo Fisher Scientific, Bremen) coupled to an EASY-nLC 1000 (Thermo Fisher Scientific). Peptides were loaded and separated at 250 nl.min^-1^ on a home-made C_18_ 50 cm capillary column picotip silica emitter tip (75 μm diameter filled with 1.9 μm Reprosil-Pur Basic C_18_-HD resin, (Dr. Maisch GmbH, Ammerbuch-Entringen, Germany)) equilibrated in solvent A (0.1% FA). Peptides were eluted using a gradient of solvent B (ACN, 0.1% FA) from 2% to 5% in 5 min, 5% to 18% in 150 min, 18% to 40% in 60 min (total length of the chromatographic run was 250 min including high ACN level steps and column regeneration). Mass spectra were acquired in data-dependent acquisition mode with the XCalibur 2.2 software (Thermo Fisher Scientific, Bremen) with automatic switching between MS and MS/MS scans using a top-10 method. MS spectra were acquired at a resolution of 70000 (at *m/z* 400) with a target value of 3 × 10^6^ ions. The scan range was limited from 300 to 1700 *m/z*. Peptide fragmentation was performed using higher-energy collision dissociation (HCD) with the energy set at 28 NCE. Intensity threshold for ions selection was set at 1 × 10^6^ ions with charge exclusion of z = 1 and z > 7. The MS/MS spectra were acquired at a resolution of 17500 (at *m/z* 400). Isolation window was set at 1.6 Th. Dynamic exclusion was employed within 45s.

#### Results analysis

Data were searched using MaxQuant (version 1.5.3.8) (with the Andromeda search engine) against a human database (20202 entries, downloaded from Uniprot the 2016.05.25), viral proteins were searched against polyprot Pf13_AHL43469 and NS protein 78788_AHL43469 and the ENV protein 78788_AHL43461.

The following search parameters were applied: carbamidomethylation of cysteines was set as a fixed modification, oxidation of methionine and protein N-terminal acetylation were set as variable modifications. The mass tolerances in MS and MS/MS were set to 5 ppm and 20 ppm respectively. Maximum peptide charge was set to 7 and 7 amino acids were required as minimum peptide length. A false discovery rate of 1% was set up for both protein and peptide levels. A match between run features was selected with a matched time window set at 1 min and alignment time window set at 20 min.

Quantification was performed using the XIC-based LFQ algorithm with the Fast LFQ mode as described in a previous publication [67]. Unique and razor peptides, included modified peptides, with at least 2 ratio count were accepted for quantification.

#### Statistical analysis of proteomics data

For the statistical analysis of one condition versus another, proteins identified in the reverse and contaminant databases and proteins only identified by site were first discarded from the list. Then, proteins exhibiting fewer than 2 LFQ values or in at least one condition were discarded from the list to avoid misidentified proteins. After log2 transformation of the leftover proteins, LFQ values were normalized by median centering within conditions (normalizeD function of the R package DAPAR [68]). Remaining proteins without any LFQ value in one condition only have been considered as proteins quantitatively present in a condition and absent in another. They have therefore been set aside and considered as differentially abundant proteins. Next, missing values were imputed using the imp.norm function of the R package norm. Proteins with a log2 (fold-change) inferior to 1 have been considered as proteins which are not significantly differentially abundant. Statistical testing of the remaining proteins (having a log2 (fold-change) superior to 1) was conducted using a limma t-test [69] thanks to the R package limma [70]. An adaptive Benjamini-Hochberg procedure was applied to the resulting p-values thanks to the function adjust.p of R package cp4p [71] using a robust method [72] to estimate the proportion of true null hypotheses among the set of statistical tests. The proteins associated to an adjusted p-value inferior to an FDR level of 1% have been considered as significantly differentially abundant proteins. Finally, the proteins of interest are therefore those which emerge from this statistical analysis supplemented by those which are considered to be absent from one condition and present in another. Statistical enrichment tests were used to highlight major biological processes that may be affected during the infection, using DAVID software [34] by selecting the set of quantified proteins among all replicates as background. Biological processes related to up/down-regulated proteins in each condition have been compared to those of up/down-regulated proteins in non-infected samples.

### Statistical analysis

If not specified, statistical analyses were performed using GraphPad Prism 6. Non-parametric Mann-Whitney tests were used to test the differences between groups. P-values of less than 0.05 were considered significant.

## Supporting information

S1 Fig Proteomic changes in ZIKV infected myoblasts and reproducibility of triplicates. A: Similar numbers of proteins (around 4000) are identified in all samples whatever the condition. A large overlap in the protein composition was observed among samples of the same condition, what shows the good reproducibility of the experiments in term of identification of proteins. B: Pairwise correlation matrix: the Pearson correlation coefficients between each pair of samples was computed using all complete pairs of LFQ intensity values measured in these samples. Because strong correlations are observed (minimum of 0.981) between all the samples, it shows a strong reproducibility of experiments in term of quantification of proteins. C: Hierarchical clustering of the samples using the Ward method and a Jaccard index based distance after replacing missing values by 1 and observed values by 0. This classification shows that the samples belonging to the same condition are grouped together which means that samples of the same condition have missing values located generally at the same protein, and that these sets of proteins with missing values are different between conditions.
(TIFF)

S2 Fig Volcano plot graphs representing proteins differentially abundant between biological conditions. Differential analyzes between human primary myoblasts infected with Pf13 or Sen91 Zika strains showing proteins differentially abundant. Each protein (represented as a dot) was mapped according to its log_2_ (fold change) on the abscissa axis and its–log_10_ (*t*-test *p*-value) on the ordinate axis. The proteins associated to an adjusted p-value inferior to an FDR level of 1% have been considered as significantly differentially abundant proteins. Pf13 versus Sen91 human primary myoblasts 24h and 48h post-infection (A, B). Mock-infected 24h post-infection versus Mock-infected 48h post-infection human primary myoblasts (C). Red—proteins that are significantly less abundant in infected condition compared to mock-infected condition. Green—proteins that are significantly more abundant in infected condition compared to mock-infected condition. Grey—proteins that do not satisfy the fold-change and FDR cutoff.
(TIFF)

S3 Fig List of modulated proteins. Dark red—proteins that are absent in infected condition and present mock-infected condition. Light red—proteins that are significantly less abundant in infected condition compared to mock-infected condition. Dark green—proteins that are present in infected condition and absent in mock-infected condition Light green—proteins that are significantly more abundant in infected condition compared to mock-infected condition.
(XLSX)
