# Supplementary figures and images for: Differentiation-dependent susceptibility of human muscle cells to Zika virus infection
Source: PLoS Negl Trop Dis. 2020 Aug 20;14(8):e0008282. doi: 10.1371/journal.pntd.0008282 (PMC7508361; doi:10.1371/journal.pntd.0008282)

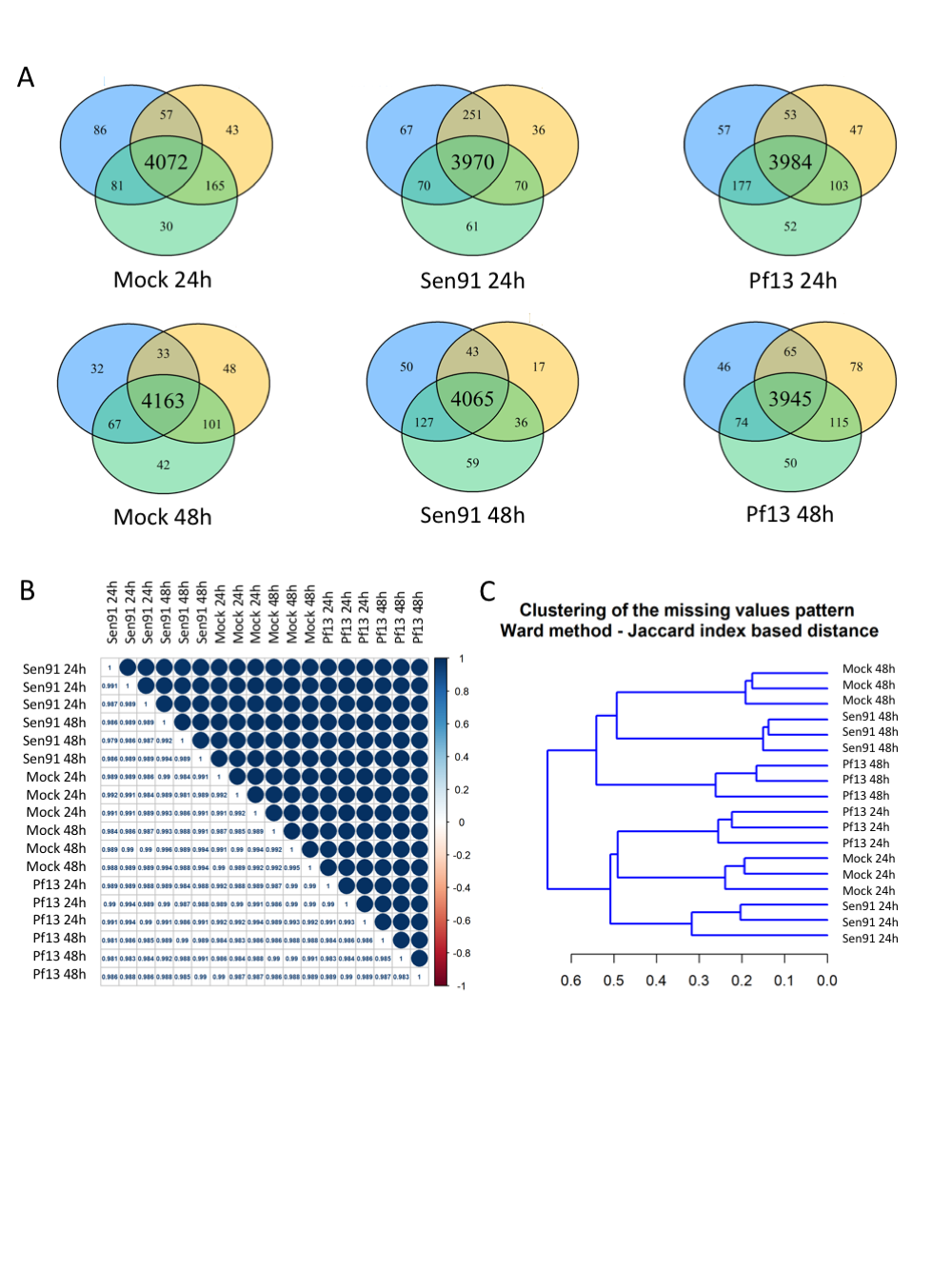

Supplement: S1 Fig — A: Similar numbers of proteins (around 4000) are identified in all samples whatever the condition. A large overlap in the protein composition was observed among samples of the same condition, what shows the good reproducibility of the experiments in term of identification of proteins. B: Pairwise correlation matrix: the Pearson correlation coefficients between each pair of samples was computed using all complete pairs of LFQ intensity values measured in these samples. Because strong correlations are observed (minimum of 0.981) between all the samples, it shows a strong reproducibility of experiments in term of quantification of proteins. C: Hierarchical clustering of the samples using the Ward method and a Jaccard index based distance after replacing missing values by 1 and observed values by 0. This classification shows that the samples belonging to the same condition are grouped together which means that samples of the same condition have missing values located generally at the same protein, and that these sets of proteins with missing values are different between conditions. (TIFF) [file pntd.0008282.s001.tiff]

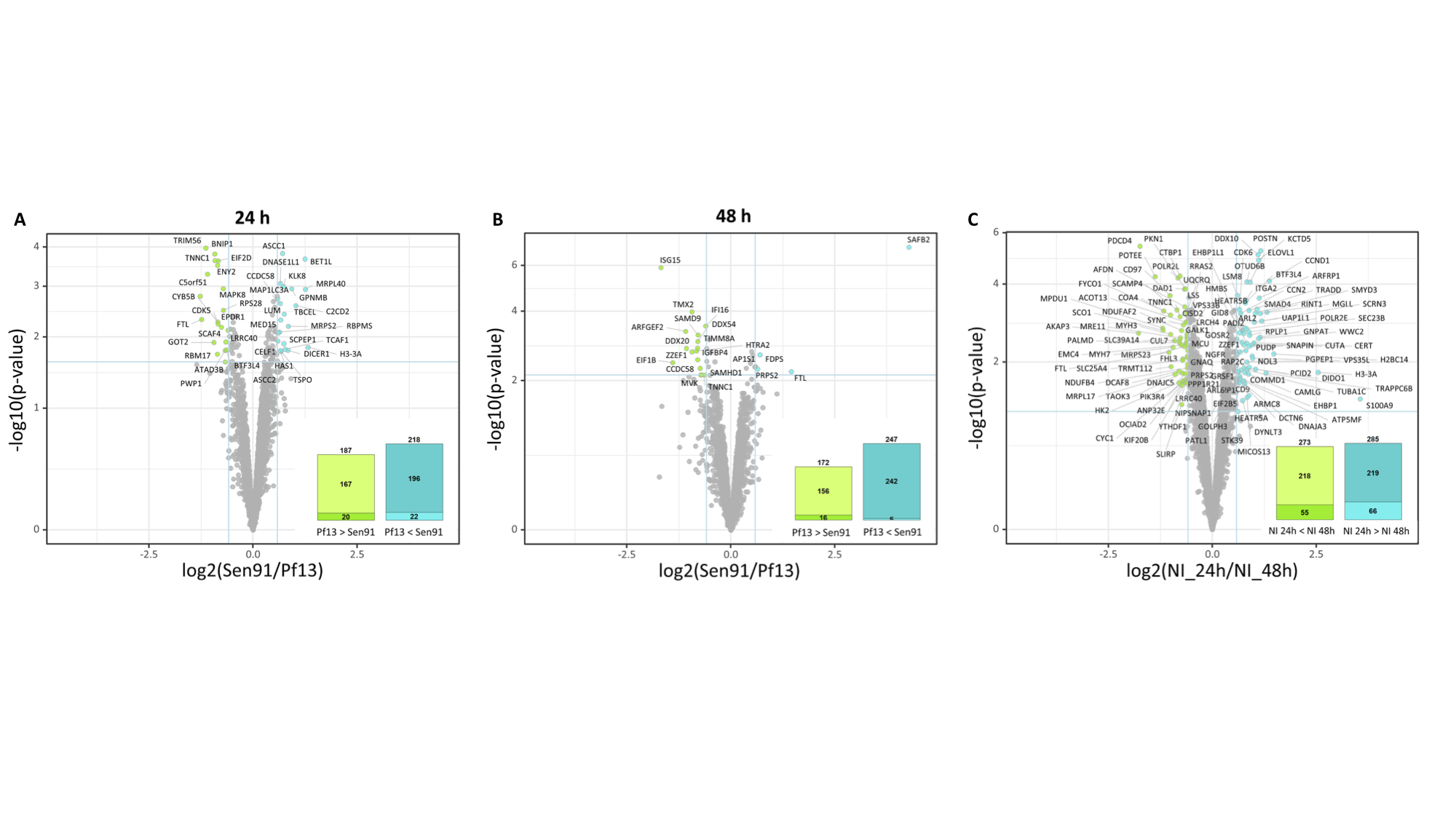

Supplement: S2 Fig — Differential analyzes between human primary myoblasts infected with Pf13 or Sen91 Zika strains showing proteins differentially abundant. Each protein (represented as a dot) was mapped according to its log2 (fold change) on the abscissa axis and its–log10 (t-test p-value) on the ordinate axis. The proteins associated to an adjusted p-value inferior to an FDR level of 1% have been considered as significantly differentially abundant proteins. Pf13 versus Sen91 human primary myoblasts 24h and 48h post-infection (A, B). Mock-infected 24h post-infection versus Mock-infected 48h post-infection human primary myoblasts (C). Red—proteins that are significantly less abundant in infected condition compared to mock-infected condition. Green—proteins that are significantly more abundant in infected condition compared to mock-infected condition. Grey—proteins that do not satisfy the fold-change and FDR cutoff. (TIFF) [file pntd.0008282.s002.tiff]
